# Clinical decision making in healthcare: the importance of best evidence

**DOI:** 10.1590/1677-5449.202401302

**Published:** 2025-03-24

**Authors:** Ronald Luiz Gomes Flumignan, Renato Delascio Lopes

**Affiliations:** 1 Universidade Federal de São Paulo – UNIFESP, São Paulo, SP, Brasil.; 2 Duke University, Durham, NC, EUA.

In a complex and constantly evolving world, medicine faces the challenge of making complex decisions to ensure the best possible care for patients. Evidence-based medicine uses scientific research data to guide the best treatment decisions for patients. In this scenario, searching for robust scientific evidence is crucial to support medical decision-making and guarantee the quality of care. Randomized controlled trials (RCTs) and systematic reviews (SRs) of RCTs constitute the ideal primary and secondary study designs, respectively, for generating reliable evidence on the effects of treatments and for synthesizing this evidence critically and pragmatically, providing essential support for clinical decision-making.^1,2^ Decision-making should be no different in vascular surgery and must also be based on evidence derived from rigorous scientific studies, such as SRs of RCTs and, when these are not available, from prospective observational studies that provide robust data on the efficacy and safety of different procedures and interventions. By incorporating this information into their practice, vascular surgeons ensure their patients receive the best available treatments, optimizing outcomes and minimizing risks.

Fanaroff et al.^3^ investigated the quality of the scientific evidence supporting the recommendations contained in the leading international cardiology guidelines. Ideally, such recommendations should be based on SRs of RCTs, but the study actually found that: 1) only a small percentage (around 8 to 14%) of the recommendations are supported by evidence from multiple RCTs; 2) a majority of the recommendations are based on observational studies (moderate evidence) or only on expert opinions (weak evidence); and 3) the proportion of recommendations supported by substantial evidence did not significantly increase between 2008 and 2018. A methodological quality analysis of carotid disease treatment guidelines found that methodological quality was high, although the degree of certainty was not high for most evidence.^4^ This means that many recommendations for cardiovascular care are not based on the most substantial scientific evidence.

Some advocate that common sense and clinical observation are preferable methods for generating evidence (“real world” evidence), questioning the external validity of traditional RCTs. However, historical examples demonstrate how well-conducted RCTs often contradict practices based on common sense and clinical observation, due to failure to understand pathophysiology, biases, and unmeasured confounding in observational studies, and to the difficulty of assessing the risks and benefits of treatments in complex health systems.^5^ Concerns about the external validity of traditional RCTs, in conjunction with the wide availability of real-world data and advanced data analysis tools, have led to claims that common sense and clinical observation, rather than RCTs, should be the preferred methods for generating evidence to support clinical decision-making. Although RCTs have been the gold standard for decades, their conclusions do not always reflect real-world clinical practice. This discrepancy arises from an incomplete understanding of pathophysiology, biases in observational studies, and failure to capture the subtle risks and benefits of treatments in complex systems like the human body.^5^

The lack of high-quality evidence and lack of robust RCTs hinders the development of strong recommendations in clinical guidelines, resulting in the provision of variable, suboptimal, and overly expensive care to patients.

One classic example of the importance of evidence-based decisions in cardiovascular medicine was the clinical observation/common sense conclusion that revascularizing multiple coronary arteries would increase mortality in patients with myocardial infarction with completely occluded vessels (ST-segment elevation myocardial infarction [STEMI]). However, after randomized trials such as the Complete vs. Culprit-Only Revascularization Strategies to Treat Multivessel Disease after Early PCI for STEMI (COMPLETE), reductions were observed in mortality and myocardial infarction rates after complete revascularization, compared to after revascularization of the artery responsible for the clinical event only. The erroneous interpretation based on clinical observation was likely due to selection bias in patients who were not randomized.^5^

The situation in vascular surgery is no different, as in the case of patients with extracranial carotid stenosis and risk of stroke or death. Carotid revascularization, whether by endarterectomy or stenting, aims to reduce the risk of stroke, one of the leading causes of death and disability worldwide. In the past, the decision to perform carotid endarterectomy was often taken based on clinical criteria, such as the presence of transient neurological symptoms (transient ischemic attacks, TIAs) or detection of severe carotid stenosis. However, more recent research showed that taking decisions based solely on clinical criteria did not identify all patients who would benefit from surgery. As research progressed, RCTs compared carotid endarterectomy with medical treatment in patients with different stroke risk profiles. These studies yielded robust evidence that surgery is more effective than medical treatment in preventing stroke in patients with symptomatic or high-grade asymptomatic carotid stenosis.^6-8^ Some common sense or observational study data suggest little or no difference between endovascular treatment and endarterectomy.^9^ However, analysis of data from randomized studies and, even more so, the high-quality systematic scrutiny of Cochrane reviews, shows that stenting for symptomatic carotid stenosis is associated with a higher risk of stroke or periprocedural death than endarterectomy. The extra risk is mainly attributable to an increased rate of mild and non-disabling strokes in people over 70 years of age. In other words, contrary to what common sense suggested, in these circumstances, it would be better to treat patients with open surgery than with endovascular techniques, even beyond 70 years of age.^10^

Incorporation of this evidence into clinical practice has led to a significant change in the indications for carotid endarterectomy. Nowadays, the decision to perform surgery is based on careful assessment of each patient’s risks and benefits, considering factors such as age, comorbidities, severity of carotid stenosis, and history of stroke or TIAs.^11^ This example illustrates how evidence-based decisions have improved carotid endarterectomy outcomes, reduced stroke risk and improved patients' quality of life.

Another landmark example of higher quality evidence changing a premise based on common sense can be seen in the case of anticoagulation of severe COVID-19 patients. Purely observational data showed high rates of vascular complications in COVID-19 patients, with thrombosis prevalence rates ranging from 20 to 40% and around 80% of these thrombosis cases being venous.^12,13^ These patients had a high mortality rate, which was often linked to complications of thrombosis, such as pulmonary embolism.^14,15^ In response, initially, and primarily at the height of the pandemic, it was concluded that higher doses of anticoagulants would be beneficial, even without confirmed thrombosis.^16^ However, the AntiCoagulaTlon cOroNavirus (ACTION) RCT found no significant difference in mortality between high and low doses.^17^ Later, a Cochrane review conducted a meta-analysis of this and other RCTs and, with high certainty of evidence, confirmed the lack of benefit in terms of mortality and warned of the increased risk of bleeding with higher doses of anticoagulants.^18^ This paradigm change demonstrates the importance of using the best available evidence to make clinical decisions.

Notwithstanding all of the advantages listed above, traditional RCTs still have some limitations, such as high cost and complexity. Individual identification of patients, face-to-face recruitment and follow-up, and the need for specific infrastructure significantly increase the cost of studies and the time needed to conduct them. Pharmaceutical industry funding is crucial to enable new technologies. Most RCTs evaluating clinical outcomes are therefore funded by pharmaceutical and medical device companies, but this can lead to prioritization of new products rather than comparing existing options. Still, some knowledge gaps remain. Many studies comparing treatment strategies, health service interventions, and the efficacy of drugs and devices that have already been approved, studies conducting assessments of therapeutic combinations, and studies to reduce the use, duration, or dose of treatments are not conducted, leaving gaps in the knowledge base.

While there is a lack of high-certainty evidence from high-quality primary and secondary studies, we must resort to appropriate strategies to optimize the execution of high-quality primary (RCTs) and secondary (SRs) studies rather than resorting to lower-quality evidence. Several initiatives have been proposed for modernization of RCTs to overcome these limitations and generate more high-quality evidence:^19^

Innovative designs: techniques such as adaptive designs, Bayesian statistics, and new composite endpoints can reduce sample sizes and costs.
Combined phases: combining trial phases II and III can optimize the process.
Registry-based RCTs: repurposing data collected for quality improvement purposes or administrative data for use in research, reducing costs and complexity.
Virtual trials: remote recruitment and follow-up of patients, facilitating and reducing the cost of participation.

By combining randomization with real-world data, we can overcome several limitations of traditional RCTs and gain more accurate insights into the efficacy of treatments in practical settings. This paradigm shift will guide development of best practices and care for patients, building a healthier future for all.

Notwithstanding the valid criticisms, randomization remains crucial for determining causality. Instead of abandoning it, we must invest in hybrid methods that apply it to real-world data. This innovative approach will enable us to build a more robust and trustworthy evidence base, driving progress in vascular medicine and beyond.

Even the secondary studies, with or without meta-analysis, that ultimately analyze risk of bias in primary studies and provide us with the degree of certainty of their evidence can also be optimized in specific scenarios. Cochrane is known worldwide for its methodologically rigorous and high-quality systematic reviews and has produced many relevant reviews on various topics, including the COVID-19 pandemic.^20^ However, a systematic review including a large number of studies can be a challenge, since it takes some time to complete all of the traditional methodological steps.^21^ Even in these cases, there are ways of optimizing resources to produce relevant studies in a shorter time frame, conducting so-called “rapid reviews”,^22^ combining elements such as: 1) interinstitutional and international collaboration, 2) acceleration of some steps using electronic tools for online study selection, and 3) speeding up steps in the publication phase.

One good example of such resource optimization occurred during the COVID-19 pandemic. Flumignan et al.^18,23^ collaborated with teams from Brazil, Lebanon, and Australia to answer what was an essential question at the time and published the first version of a rapid review about 4 months after the electronic searches were conducted, which was co-published in high impact journals,^24^ then updated and published again shortly afterwards, this time with high certainty of evidence.^18^ The study evidence was also presented in podcasts and translated into more than 10 different languages, to improve decision-making worldwide.^25,26^ Whereas some traditional systematic reviews can take from 2 to 10 years from registration to final publication, in a few months this rapid review was able to guide decision making all over the globe.

The quest for better evidence must be a constant commitment for health professionals. This means: 1) keeping up to date on the latest research and clinical guidelines; 2) critically evaluating the quality of the information found; 3) prioritizing studies with robust methodologies and high levels of evidence, i.e., those less subject to risk of bias; 4) considering the individual characteristics of patients and the clinical context; and 5) being flexible and willing to change opinions when new evidence is presented.

By adopting an evidence-based culture, medicine can ensure that decisions taken are more often correct and are safe and effective, with direct benefits for patient health. It is important to point out that the search for best evidence is no substitute for clinical experience, common sense, and a humanistic spirit, which must be a part of all medical care. Healthcare professionals must always consider the individual circumstances of each case and use their knowledge to take the best decision for each patient. On the other hand, common sense alone can induce us to make dramatic errors if we do not avail ourselves of the best of what is available when taking clinical decisions in conjunction with the patient.

We live in a time when medicine is evidence-based, and this is a dynamic and continuous process. Health professionals, educational and research institutions, and governments all must work together to foster a culture of research and publication of high quality scientific knowledge. Investing in the best evidence, we are investing in more effective and safer medicine for all. This is not a matter of belief, since there are no arguments against facts. Instead, it is a matter of practicing what is best for the patient’s benefit.

## References

[1] Cumpston M, Flemyng E, Thomas J, Higgins JPT, Deeks JJ, Clarke MJ, Higgins JPT, Thomas J, Chandler J, Cumpston M, Li T, Page MJ (2023). Cochrane handbook for systematic reviews of interventions version 6.4 (updated august 2023).

[2] Flumignan RLG, Nakano LCU, Amorim JED, Atallah ÁN (2024). “2023 ISTH update of the 2022 ISTH guidelines for antithrombotic treatment in COVID-19”: comment from Flumignan et al. J Thromb Haemost.

[3] Fanaroff AC, Califf RM, Windecker S, Smith SC, Lopes RD (2019). Levels of Evidence Supporting American College of Cardiology/American Heart Association and European Society of Cardiology Guidelines, 2008-2018. JAMA.

[4] Coutinho SGB, Ricardo JC, Coutinho AIM, Cavalcante LP (2022). Qualidade metodológica das diretrizes de tratamento da doença arterial obstrutiva carotídea: uma avaliação sistemática com a utilização do instrumento AGREE II. J Vasc Bras.

[5] Fanaroff AC, Califf RM, Harrington RA (2020). Randomized trials versus common sense and clinical observation. J Am Coll Cardiol.

[6] North American Symptomatic Carotid Endarterectomy Trial Collaborators (1991). Beneficial effect of carotid endarterectomy in symptomatic patients with high-grade carotid stenosis. N Engl J Med.

[7] Walker MD (1995). Endarterectomy for asymptomatic carotid artery stenosis. Executive Committee for the Asymptomatic Carotid Atherosclerosis Study. JAMA.

[8] Aboyans V, Ricco JB, Bartelink MLEL (2018). Guidelines on the Diagnosis and Treatment of Peripheral Arterial Diseases, in collaboration with the European Society for Vascular Surgery (ESVS). Eur J Vasc Endovasc Surg Off J Eur Soc Vasc Surg..

[9] Oliveira TF, Centellas CDR, Dalio MB, Joviliano EE (2024). Short term outcomes of carotid surgery: the real-world experience of a single teaching center. J Vasc Bras.

[10] Müller MD, Lyrer P, Brown MM, Bonati LH (2020). Carotid artery stenting versus endarterectomy for treatment of carotid artery stenosis. Cochrane Database Syst Rev.

[11] Naylor R, Rantner B, Ancetti S (2023). Editor’s Choice – European Society for Vascular Surgery (ESVS) 2023 Clinical Practice Guidelines on the Management of Atherosclerotic Carotid and Vertebral Artery Disease. Eur J Vasc Endovasc Surg.

[12] Correia RM, Santos BC, Carvalho AAG (2022). Vascular complications in 305 severely ill patients with COVID-19: a cohort study. Sao Paulo Med J.

[13] Benson RA, Nandhra S (2021). Outcomes of vascular and endovascular interventions performed during the coronavirus disease 2019 (COVID-19) pandemic. Ann Surg.

[14] COVIDSurg Collaborative (2021). Timing of surgery following SARS-CoV-2 infection: an international prospective cohort study. Anaesthesia.

[15] COVIDSurg Collaborative (2022). SARS-CoV-2 infection and venous thromboembolism after surgery: an international prospective cohort study. Anaesthesia.

[16] Sobreira ML, Marques MA (2020). Anticoagulants as panacea in COVID-19 infection. J Vasc Bras.

[17] Lopes RD, Barros E, Silva PGM (2021). Therapeutic versus prophylactic anticoagulation for patients admitted to hospital with COVID-19 and elevated D-dimer concentration (ACTION): an open-label, multicentre, randomised, controlled trial. Lancet.

[18] Flumignan RL, Civile VT, Tinôco JDS (2022). Anticoagulants for people hospitalised with COVID-19. Cochrane Database Syst Rev.

[19] Fanaroff AC, Califf RM, Lopes RD (2020). New approaches to conducting randomized controlled trials. J Am Coll Cardiol.

[20] Cochrane Library (2024). COVID-19 resources.

[21] Flumignan RL, Trevisani VF, Lopes RD, Baptista-Silva JC, Flumignan CD, Nakano LC (2021). Ultrasound guidance for arterial (other than femoral) catheterisation in adults. Cochrane Database Syst Rev.

[22] Cochrane (2020). Cochrane COVID Rapid Reviews.

[23] Flumignan RL, Tinôco JDS, Pascoal PI (2020). Prophylactic anticoagulants for people hospitalised with COVID-19. Cochrane Database Syst Rev.

[24] Flumignan RL, Tinôco JDS, Pascoal PI (2021). Prophylactic anticoagulants for people hospitalized with COVID-19: systematic review. Br J Surg.

[25] Flumignan RL, Tinôco JD S, Pascoal PI (2020). Prophylactic anticoagulants for people hospitalised with COVID‐19.

[26] Flumignan RL, Tinôco JD S, Pascoal PI (2020). Prophylactic anticoagulants for people hospitalised with COVID‐19.

