# Supplementary material for: Clinical decision making in healthcare: the importance of best evidence
Source: J Vasc Bras. 2025 Mar 24;24:e20240130. doi: 10.1590/1677-5449.202401302 (PMC11938930; doi:10.1590/1677-5449.202401302)
Supplement: Translation

# Translation: Tomada de decisão clínica em saúde: a importância das melhores evidências

**DOI:** 10.1590/1677-5449.202401301

**Authors:** Ronald Luiz Gomes Flumignan, Renato Delascio Lopes

**Affiliations:** 1 Universidade Federal de São Paulo – UNIFESP, São Paulo, SP, Brasil.; 2 Duke University, Durham, NC, EUA.

Em um mundo complexo e em constante evolução, a medicina enfrenta o desafio de tomar decisões complexas com o objetivo de garantir o melhor cuidado possível para seus pacientes. A medicina baseada em evidências busca utilizar dados de pesquisas científicas para orientar as melhores decisões de tratamento para os pacientes. Nesse contexto, a busca por evidências científicas robustas se torna crucial para embasar as escolhas médicas e garantir a qualidade da assistência prestada. Os ensaios clínicos randomizados (ECRs) e as revisões sistemáticas (RSs) de ECRs se destacam, respectivamente, como o método ideal de estudos primários e secundários para gerar evidências confiáveis sobre os efeitos de tratamentos e uma síntese dessas evidências de maneira crítica e pragmática, sendo fundamentais para a tomada de decisão clínica^1,2^ . Na cirurgia vascular, as decisões não deveriam ser diferentes, mas também baseadas em evidências que se fundamentam em pesquisas científicas rigorosas, como RSs de ECRs e, em caso de ausência desses, em estudos observacionais prospectivos, que fornecem dados robustos sobre a efetividade e a segurança de diferentes procedimentos e intervenções. Ao incorporar essas informações em sua prática, os cirurgiões vasculares garantem que seus pacientes recebam os melhores tratamentos disponíveis, otimizando resultados e minimizando riscos.

Fanaroff et al.^3^ investigaram a qualidade da base científica por trás das recomendações feitas pelas principais diretrizes mundiais de cardiologia. Idealmente, essas recomendações deveriam ser baseadas em RSs de ECRs, mas o estudo descobriu que: 1) apenas uma pequena porcentagem (cerca de 8 a 14%) das recomendações se apoia em evidências de múltiplos ECRs; 2) a maioria das recomendações baseiam-se em estudos observacionais (evidência moderada) ou apenas em opiniões de especialistas (evidência fraca); 3) a proporção de recomendações apoiadas por evidências fortes não aumentou significativamente entre 2008 e 2018. Em uma análise de qualidade metodológica sobre diretrizes de tratamento de doença carotídea, foi evidenciada uma alta qualidade metodológica, embora a maioria das evidências ainda não sejam de alta certeza^4^ . Isso significa que muitas recomendações para cuidados em medicina cardiovascular não são baseadas nas evidências científicas mais fortes.

Alguns defendem o uso do senso comum e da observação clínica como métodos preferíveis para gerar evidências (as evidências do “mundo real”), questionando a validade externa dos ECRs tradicionais. No entanto, exemplos históricos demonstram como os ECRs bem realizados frequentemente contradizem práticas baseadas no senso comum e na observação clínica, devido à incompreensão da fisiopatologia, dos vieses e da confusão não medida em pesquisas observacionais, e à dificuldade de avaliar os riscos e benefícios dos tratamentos em sistemas de saúde complexos^5^ . As preocupações sobre a validade externa dos ECRs tradicionais, juntamente com a ampla disponibilidade de dados do mundo real e de ferramentas avançadas de análise de dados, levaram a alegações de que o bom senso e a observação clínica, em vez dos ECRs, deveriam ser o método preferido para gerar evidências que apoiem a tomada de decisão clínica. Embora os ECRs tenham sido o padrão-ouro por décadas, suas conclusões nem sempre refletem a prática clínica real. Essa discrepância surge da incompleta compreensão da fisiopatologia, de vieses em pesquisas observacionais e da falha em captar os riscos e benefícios sutis dos tratamentos em sistemas complexos como o corpo humano^5^ .

A falta de evidências de alta qualidade, assim como a falta de ECRs robustos, impedem o desenvolvimento de recomendações fortes nas diretrizes clínicas, levando a cuidados variáveis, subótimos e excessivamente caros para os pacientes.

Um exemplo clássico da importância das decisões baseadas em evidências na medicina cardiovascular foi a observação clínica/senso comum de que revascularizar múltiplas coronárias em pacientes com infarto do miocárdio com vasos completamente ocluídos (infarto do miocárdio com elevação do segmento ST) aumentaria a mortalidade. Entretanto, após estudos randomizados como o *Complete versus Culprit-Only Revascularization Strategies to Treat Multivessel Disease after Early PCI for STEMI* (COMPLETE), foi evidenciada a redução das taxas de mortalidade e infarto do miocárdio com a revascularização completa, quando comparada com a revascularização apenas da artéria responsável pelo evento clínico. É provável que a interpretação errônea baseada na observação clínica se deu por viés de seleção nos pacientes que não foram randomizados^5^ .

Na cirurgia vascular não é diferente, como por exemplo no caso dos pacientes com estenose carotídea extracraniana e risco de acidente vascular cerebral (AVC) ou morte. A revascularização carotídea, seja por endarterectomia ou colocação de stent, visa reduzir o AVC, uma das principais causas de morte e incapacidade no mundo. No passado, a endarterectomia carotídea era frequentemente realizada com base em critérios clínicos, como a presença de sintomas neurológicos transitórios (ataques isquêmicos transitórios, AITs) ou a detecção de estenose carotídea grave. No entanto, pesquisas subsequentes demonstraram que a tomada de decisão baseada apenas em critérios clínicos não era suficiente para identificar todos os pacientes que se beneficiariam da cirurgia. Com o avanço das pesquisas, ECRs compararam a endarterectomia carotídea com o tratamento medicamentoso em pacientes com diferentes perfis de risco de AVC. Esses estudos forneceram evidências robustas de que a cirurgia é mais eficaz do que o tratamento medicamentoso na prevenção de AVC em pacientes com estenose carotídea sintomática ou assintomática de alto grau^6-8^ . Alguns dados de senso comum ou estudos observacionais podem sugerir que há pouca ou nenhuma diferença entre o tratamento endovascular e a endarterectomia^9^ . Entretanto, quando analisamos dados de estudos randomizados e mais ainda após um escrutínio sistemático de alta qualidade das revisões Cochrane, o implante de stent para estenose carotídea sintomática está associado a um risco maior de AVC ou de morte periprocedimento do que a endarterectomia. Esse risco extra é atribuído principalmente a um aumento de AVCs leves e não incapacitantes que ocorrem em pessoas com mais de 70 anos. Ou seja, ao contrário do que apontava o senso comum, seria melhor submeter um paciente a cirurgia aberta que endovascular, mesmo após os 70 anos de idade, nessas condições apontadas^10^ .

A incorporação dessas evidências na prática clínica levou a uma mudança significativa na forma como a endarterectomia carotídea é indicada. Hoje em dia, a decisão de realizar a cirurgia é baseada em uma avaliação criteriosa dos riscos e benefícios individuais de cada paciente, considerando fatores como idade, comorbidades, gravidade da estenose carotídea e histórico de AVC ou AITs^11^ . Esse exemplo ilustra como as decisões baseadas em evidências contribuíram para a melhoria dos resultados na endarterectomia carotídea, reduzindo o risco de AVC e melhorando a qualidade de vida dos pacientes.

Outro exemplo emblemático em que as evidências de melhor qualidade mudaram o postulado pelo senso comum foi no caso da anticoagulação em pacientes graves com COVID-19. Alguns dados puramente observacionais mostraram uma taxa de complicação vascular alta em pacientes com COVID-19, com prevalências de trombose de cerca de 20 a 40% e cerca de 80% dos casos de trombose sendo venosa^12,13^ . Esses pacientes tiveram uma alta taxa de mortalidade, que muitas vezes foi associada a complicações dessa trombose, como a embolia pulmonar^14,15^ . Assim, inicialmente, principalmente no auge da pandemia, acreditava-se que doses mais altas de anticoagulantes seriam benéficas, mesmo sem confirmação de trombose^16^ . O ECR *AntiCoagulaTlon cOroNavirus* (ACTION), no entanto, não encontrou diferença significativa em termos de mortalidade entre doses altas e baixas^17^ . Posteriormente, uma revisão Cochrane realizou uma metanálise desse e de outros ECRs e, com alta certeza de evidência, confirmou a falta de benefício em termos de mortalidade e alertou para o aumento do risco de sangramento com doses mais altas de anticoagulantes^18^ . Essa mudança de paradigma demonstra a importância de utilizar as melhores evidências disponíveis para tomar decisões clínicas.

Apesar de todas as vantagens apresentadas acima, os ECRs tradicionais apresentam algumas limitações, como o alto custo e a complexidade. A identificação individual de pacientes, o recrutamento e acompanhamento presenciais, e a necessidade de infraestrutura específica aumentam significativamente o custo e o tempo de execução dos estudos. O financiamento da indústria farmacêutica é determinante para viabilizar novas tecnologias. Assim, a maioria dos ECRs que avaliam resultados clínicos são financiados por empresas farmacêuticas e de dispositivos médicos, mas isso pode levar à priorização de novos produtos em detrimento da comparação de opções existentes. Ainda assim, algumas lacunas no conhecimento persistem. Muitos estudos comparativos de estratégias de tratamento, intervenções em serviços de saúde, eficácia de medicamentos e dispositivos aprovados, avaliações de combinações terapêuticas e estudos para reduzir o uso, a duração ou a dose de um tratamento não são realizados, deixando lacunas na base de conhecimento.

Embora exista uma carência de evidências de alta certeza provenientes de estudos primários e secundários de boa qualidade, devemos recorrer a estratégias adequadas para otimizar a execução de estudos primários (ECRs) e secundários (RSs) de alta qualidade e não recorrer a evidências com qualidade mais baixa. Para superar essas limitações e gerar mais evidências de alta qualidade, diversas iniciativas propõem a modernização dos ECRs^19^ :

Designs inovadores: técnicas como designs adaptativos, estatísticas bayesianas e novos *endpoints* compostos podem reduzir o tamanho da amostra e os custos.
Fases combinadas: combinar as fases II e III dos estudos pode otimizar o processo.
ECRs baseados em registros: aproveitar dados coletados para fins de aprimoramento da qualidade ou dados administrativos para pesquisas, diminuindo custos e complexidade.
Ensaios virtuais: recrutamento e acompanhamento remoto dos pacientes, facilitando e barateando a participação.

Ao agregar a randomização com dados do mundo real, podemos superar várias limitações dos ECRs tradicionais e obter *insights* mais precisos sobre a efetividade dos tratamentos em cenários práticos. Essa mudança de paradigma guiará o desenvolvimento de melhores práticas e cuidados para os pacientes, construindo um futuro mais saudável para todos.

Apesar das críticas válidas, a randomização continua sendo crucial para determinar a causalidade. Em vez de abandoná-la, devemos investir em métodos híbridos que a apliquem a dados do mundo real. Essa abordagem inovadora nos permitirá construir uma base de evidências mais robusta e confiável, impulsionando o progresso na medicina vascular e em geral.

Os estudos secundários com ou sem metanálise que finalmente analisam o risco de viés dos estudos primários e nos fornecem a certeza da evidência também podem ser otimizados em cenários específicos. A Cochrane é conhecida globalmente por suas revisões sistemáticas metodologicamente rigorosas e de alta qualidade e já produziu inúmeras revisões relevantes sobre diversos temas, inclusive a pandemia de COVID-19^20^ . No entanto, uma revisão sistemática incluindo um número grande de estudos pode ser desafiadora, pois leva certo tempo a ser concluída para seguir todos os passos metodológicos tradicionais^21^ . Mesmo nesses casos há a possibilidade de otimizar recursos para se produzir estudos relevantes num tempo mais curto, as chamadas “revisões rápidas”^22^ , associando alguns fatores como: 1) colaborar interinstitucional e internacionalmente, 2) acelerar passos com recursos eletrônicos de seleção de estudos on-line, 3) passos editoriais mais céleres.

Um bom exemplo dessa otimização de recursos foi durante a pandemia de COVID-19. Flumignan et al.^18,23^ colaboraram com grupos do Brasil, do Líbano e da Austrália para responder a uma pergunta essencial na época, publicaram a primeira versão da revisão rápida cerca de 4 meses após as buscas eletrônicas, que foi copublicada em revistas de alta relevância^24^ , atualizada e publicada novamente pouco depois, agora com evidências de alta certeza^18^ . Além disso, suas evidências foram transformadas em podcasts e traduzidas para mais de 10 línguas diferentes, para a melhor tomada de decisão em todo mundo^25,26^ . Enquanto algumas revisões sistemáticas tradicionais demoram cerca de 2 a 10 anos entre registro e publicação final, essa revisão rápida em poucos meses conseguiu orientar a tomada de decisão ao redor do globo.

A busca por melhores evidências deve ser um compromisso constante dos profissionais de saúde. Isso significa: 1) manter-se atualizado sobre as últimas pesquisas e diretrizes clínicas; 2) avaliar criticamente a qualidade das informações encontradas; 3) priorizar estudos com metodologias robustas e alto nível de evidência, ou seja, menos sujeitos a risco de viés; 4) considerar as características individuais dos pacientes e o contexto clínico; 5) ser flexível e disposto a mudar de opinião quando novas evidências forem apresentadas.

Ao adotar uma cultura baseada em evidências, a medicina pode garantir decisões mais assertivas, seguras e eficazes, beneficiando diretamente a saúde dos pacientes. É importante ressaltar que a busca por melhores evidências não substitui a experiência clínica, o bom senso e o caráter humanístico, que devem estar presentes em todos os atendimentos médicos. O profissional de saúde deve sempre considerar as particularidades de cada caso e usar seu conhecimento para tomar a melhor decisão para o paciente. Por outro lado, o senso comum isolado pode nos levar a equívocos dramáticos se não usarmos o que há de melhor disponível para tomar uma decisão clínica junto ao paciente.

Vivemos num momento em que a medicina é baseada em evidências, e isso é um processo dinâmico e contínuo. É fundamental que profissionais de saúde, instituições de ensino e pesquisa e governos trabalhem em conjunto para promover a cultura da pesquisa e a disseminação do conhecimento científico de qualidade. Ao investir em melhores evidências, investimos em uma medicina mais eficaz e segura para todos. Não é questão de acreditar, pois contra fatos não há argumentos, mas de praticar o que há de melhor em benefício do paciente.
